# Telomerase (*hTERT*) Overexpression Reveals a Promising Prognostic Biomarker and Therapeutical Target in Different Clinical Subtypes of Pediatric Acute Lymphoblastic Leukaemia

**DOI:** 10.3390/genes12101632

**Published:** 2021-10-17

**Authors:** Beatriz Maria Dias Nogueira, Laudreísa da Costa Pantoja, Emerson Lucena da Silva, Fernando Augusto Rodrigues Mello Júnior, Eliel Barbosa Teixeira, Alayde Vieira Wanderley, Jersey Heitor da Silva Maués, Manoel Odorico de Moraes Filho, Maria Elisabete Amaral de Moraes, Raquel Carvalho Montenegro, André Salim Khayat, Caroline Aquino Moreira-Nunes

**Affiliations:** 1 Pharmacogenetics Laboratory, Drug Research and Development Center (NPDM), Department of Medicine, Federal University of Ceará, Fortaleza, CE 60430-275, Brazil; bmdnogueira@gmail.com (B.M.D.N.); lucenaemerson@alu.ufc.br (E.L.d.S.); odorico@ufc.br (M.O.d.M.F.); betemora@ufc.br (M.E.A.d.M.); rcm.montenegro@gmail.com (R.C.M.); 2 Department of Pediatrics, Octávio Lobo Children’s Hospital, Belém, PA 60430-275, Brazil; laudreisa@hotmail.com (L.d.C.P.); alaydevieira@yahoo.com.br (A.V.W.); 3 Molecular Biology Laboratory, Ophir Loyola Hospital, Belém, PA 66063-240, Brazil; fernando.mellojr@hotmail.com; 4 Oncology Research Center, Department of Biological Sciences, Federal University of Pará, Belém, PA 66073-005, Brazil; elielcn2015@gmail.com (E.B.T.); khayatas@gmail.com (A.S.K.); 5 Hematology and Transfusion Medicine Center, University of Campinas, Campinas, SP 13083-970, Brazil; jerseymaues@gmail.com

**Keywords:** acute lymphoblastic leukemia, gene expression, molecular biomarker, telomerase

## Abstract

Acute Lymphoblastic Leukemia (ALL) is a neoplasm of the hematopoietic system defined as a clonal expansion of an abnormal lymphoid precursor cell. It mostly affects children under five years of age and is the most common tumor to afflict pediatric patients. The expression of the human telomerase gene (*hTERT*) in patients with ALL has been studied as a biomarker and could become a new therapeutic target. We evaluate the role of *hTERT* gene expression in ALL pediatric patients, through quantitative real-time PCR technique, and the possible correlation between *hTERT* expression and clinical variables: gender, age, white blood cells (WBC), gene fusions, and immunophenotyping. The analysis between healthy controls and ALL patients (*N* = 244) was statistically significant (*p* < 0.001), demonstrating *hTERT* overexpression in these patients. In comparison with the usual set of clinical variables, the data were not statistically significant (*p* > 0.05), indicating that *hTERT* is equally overexpressed among patients regardless of gender, age, gene fusions, and immunophenotyping. Moreover, patients who presented a higher *hTERT* expression level had a significant (*p* < 0.0001) lower overall survival rate. In summary, *hTERT* expression emerges as an important molecular pathway in leukemogenesis regardless patient’s clinical variables, thus, the data here presented pointed it as a valuable biomarker in pediatric acute lymphoblastic leukemia and a promising target for new therapeutic and prognostic measures.

## 1. Introduction

Acute lymphoblastic leukemia (ALL) is a hematopoietic malignant disorder characterized by the affliction of lymphoid progenitor cells leading to their transformation and abnormal proliferation of immature clonal cells, denominated as blasts. ALL composes 80% of all neoplasia cases diagnosed in children and its survival rate demonstrates that children between 1 and 9 years with B-cell ALL tend to have a better prognosis than under 1-year-old infants and children 10 years or older, that in general presents a shorter overall survival [1,2,3,4,5,6,7,8].

During leukemogenesis onset, hematopoietic cells undergo genetic mutations that may trigger chromosomal translocations due to defects on chromosome ends, denominated as telomeres, thereby conferring their malignant phenotype [1,2,9,10,11,12]. Dysfunctions afflicting the telomere complex may expose the DNA to enzymatic activity leading to degradation, breakage, and deleterious processes on the genetic material. The main consequence of loss or dysfunction of the telomeres is a chromosomal instability that further aggravates and may lead to genetic fusions, karyotype abnormalities, cancer progression, and poor clinical outcomes [13,14].

Telomere shortening may be reverted by the human telomerase gene (*hTERT*) activity, however, this enzyme is only present in limited amounts in human cells [15,16]. Reactivation of *hTERT* was identified as a common and important biomarker in cancer, and research data demonstrates that enzyme activity plays a critical role in aberrant cell proliferation, tumor progression, and immortality [17].

The altered *hTERT* expression in patients with hematological diseases as ALL has been widely discussed in the literature as a possible molecular biomarker that could be useful in the development new targeted therapeutics for neoplastic patients [18,19,20]. Assessing altered gene expression in patients with ALL is being studied as a possible way to detect new biomarkers, which could lead to the development of new targeted drugs [19].

Thus, the aim of this study was to assess the *hTERT* expression profile in a Brazilian cohort of pediatric ALL patients, to evaluate its possible role in leukemogenesis pathway, and as a prognostic biomarker and potential novel therapeutical target.

## 2. Patients and Methods

### 2.1. Biological Samples

The present study has a total number of 244 pediatric patients diagnosed with Acute Lymphoblastic Leukemia at Octávio Lobo Children’s Hospital (Belém, PA, Brazil) according to the French American British (FAB) criteria [21]. The clinical data from patients were analyzed based on risk-stratification criteria of Berlin-Franklin-Münster (BFM) [22].

Patients’ blood samples were collected at the time of diagnosis, and samples from 10 healthy volunteers were used as control. The study was approved by the Ethics Committee of the Ophir Loyola Hospital (approval number: 2,798,615), informed written assent was obtained from the patients’ legal guardians and all methods were carried out in accordance with Helsinki guidelines and regulations.

### 2.2. Extraction of RNA and Reverse Transcription of RNA to cDNA

RNA from samples was extracted with TRIzol Reagent^®^ (Invitrogen, Walthan, MA, USA) according to the manufacturer’s instructions. From 25 µL of RNA, the cDNA was synthesized using High-Capacity cDNA Reverse Transcriptase kit (Life Technologies, Carlsbad, CA, USA) to convert the extracted and purified RNA to cDNA. The conversion step was performed on a Veriti^®^ Thermal Cycler (Applied Biosystems, Foster City, CA, USA). After this step, the samples were stored in a freezer at −20 °C until use for analysis.

### 2.3. Genetic Fusion Identification

Multiplex Polymerase Chain Reactions were developed in order to identify fused transcripts that commonly occur in cases of ALL in the studied region, such as: *E2A-PBX1*, *MLL-AF4*, *BCR-ABL*, *TEL-AML1*, and *SIL-TAL*. For this purpose, the primers are presented in Supplementary File 1 (Table S1).

Multiplex PCR was performed in a final volume of 16.5 μL with 1 μL of cDNA; 4.25 μL of nuclease free water; 6.25 μL of GoTaq Colorless Master Mix (Promega, Madison, WI, USA) and 0.5 μL of each primer. After initial denaturation at 95 °C for 3 min, 35 cycles were performed, including 94 °C for 2 min, 61 °C for 1 min, and 70 °C for 2 min in each cycle. The final extension, at 70 °C for 30 min, was performed to ensure complete elongation of all PCR products. All rounds of reactions were done along with two additional reactions. One with a c-DNA-free tube (negative control), to avoid false-positive results due to possible contamination with other genetic materials; and another with a tube containing a pair of primers with annealed in an unaltered gene (H36B4), reducing the possibility of false-negative results due to poor quality of the c-DNA sample or reagents, presence of reaction inhibitors, or human error.

#### 2.3.1. Electrophoresis in Agarose Gel

For the possible visualization and analysis of PCR products, which would characterize the occurrence of gene fusion, agarose gel electrophoresis was performed. The gel was prepared by mixing 1.5 g of powdered agarose into 100 mL of 1X TEB Buffer. After heating in a microwave until the powder had completely dissolved, 10 µL of SYBR^®^ Safe DNA Gel Stain (Life Technologies, Carlsbad, CA, USA) was added. After polymerization, the gel was placed in the electrophoresis vat, then 1L of 1X TEB buffer was added and the samples, homogenized with BlueJuiceTM Gel Loading Buffer (Life Technologies, Carlsbad, CA, USA) at a ratio of 4 to 1, were applied one to each well. In addition to the samples, 0.5 μL of 1 Kb Plus DNA Ladder (Invitrogen, Walthan, MA, USA) was applied to the first well so that the size of the amplicons obtained could be estimated. After that, the poles of the vat were connected to an electrophoresis source, submitting a current of 100 Volts for approximately 30 min. At the end of the process, the gel was placed on a Transilluminator, Safe Imager 2.0 Blue Light Transilluminator (Invitrogen, Walthan, MA, USA), for visualization and analysis of possible bands.

#### 2.3.2. Sequencing of Fusioned Fragments

To prove the existence of the fused genes detected by PCR-multiplex, the direct sequencing of the PCR product of the fused fragments was performed, serving, then, as validation of the PCR. The technique was performed in the automatic sequencer ABI Prism-3130 (Applied Biosystems, Waltham, MA, USA). The methodology used is based on the biochemical synthesis of the DNA using the Big Dye Terminator Cycle Sequencing kit (Applied Biosystem, Foster City, CA, USA). The nucleotide sequences produced were analyzed using the Sequence Scanner V 1.0 program (Applied Biosystem, Foster City, CA, USA).

### 2.4. Validation of Gene Expression by Real-Time Quantitative Polymerase Chain Reaction (qPCR)

The gene selected for evaluation of telomerase expression was *hTERT* (Hs_00972650_m1), and the genes *ABL* (Hs_01104728_m1), and *GAPDH* (Hs_02786624_g1) were used as an internal control. The detection method was the TaqMan^®^ Gene expression assays system (Applied Biosystems^®^, Foster City, CA, USA) and qPCR was performed using QuantStudio5 Real-Time PCR system (Applied Biosystems, Foster City, CA, USA).

For each sample, the following were used: 3 μL of cDNA, 1 μL of each primer/probe, 12.5 μL of TaqMan^®^ Gene Expression Master Mix (Life Technologies, Carlsbad, CA, USA), and 8.5 μL of ultra-pure water. The gene-expression levels were based on absolute and relative analyses and calculated using the 2−^ΔΔCQ^ (delta delta cycle quantification) method. Using the heath samples as the calibrator/control.

Fold change data are represented as mean ± standard deviation of three independent experiments. Each sample was analyzed in triplicate for experimental and technique validation, according to the international standards for evaluation of gene expression by real-time PCR [23,24].

### 2.5. hTERT Protein-Protein Interaction Network and Functional Enrichment Analysis

To identify the biological functions in the group of higher *hTERT* expression (>5 fold change) and the pathways involved in acute lymphoblastic leukaemia, we performed a PPI (Protein-Protein Interaction) analysis. We built the prototype of the *hTERT* PPI with Cytoscape (v3.8.0) [25], retrieving the protein data in the STRING (http://string-db.org/, accessed on 14 September 2021). We apply a confidence score of 0.70 and test a maximum number of 50 interactions to generate a core network.

Then, the Molecular Complex Detection (MCODE) tool was applied to the generated PPI network modules with maximum score [26]. The following parameters were used: degree cutoff ≥ 2, node score cutoff ≥ 2, K-core ≥ 2 and max. depth = 100. The highest scoring PPI network topology was selected and validated in the STRING database (http://string-db.org/, accessed on 15 September 2021). In the subsequent step, the functions of the PPI genes validated in GO were made in: Gene Ontology (http://geneontology.org/, accessed on 16 September 2021), REACTOME (https://reactome.org/, accessed on 16 September 2021) and KEGG (https://www. genome.jp/kegg/, accessed on 16 September 2021).

### 2.6. Statistical Analysis

Data were expressed as mean or median ± dispersion measures depending on the normality of samples. The Shapiro–Wilk test was used to evaluate the sample’s distribution. Comparison tests were performed by Analysis of Variance (ANOVA) followed by the Mann–Whitney test for comparison between two groups, and Kruskal–Wallis test and Dunn’s Multiple Comparison post-test when more than two groups were compared in non-parametric tests. To parametric analysis, t-test and Bonferroni’s post-test were used, when two and more than two groups were analyzed, respectively. The Chi-Square test was applied in order to compare the likely influence between two clinical variables.

Overall patient survival was calculated from the date of diagnosis to the date of mortality or the last follow-up using the Kaplan–Meier method with the log-rank test. All statistics were performed using R (https://www.r-project.org, accessed on 14 September 2021) [27]. Significant differences were considered with an interval of confidence of 95% (*p* < 0.05).

## 3. Results

### 3.1. Clinical Features of Acute Lymphoblastic Leukemia Patients

From a total of 244 ALL pediatric patients included in this study, only 148 presented, in their medical records, the complete clinical and epidemiologic data to perform the statistical correlations and risk management assess. The median age of diagnosis was 6.0 years (range 0.83–18), from the total patients, 84 (56.8%) were male and 64 were female (43.2%).

Patients were sorted into different groups by age (>1, 1–9, ≥10), the white blood cells count (WBC), in groups of >50 × 10^3^/mm^3^, 50–100 × 10^3^/mm^3^ and >100 × 10^3^/mm^3^, gender (male or female), according to their immunophenotype (B-ALL, T-ALL and biphenotypic) and classical genetic fusions. Patients who were participating in this study presented the following genetic fusions: *E2A-PBX1* (30), *BCR-ABL* (19), *TEL-AML1* (10), *MLL-AF4* (7), *SIL-TAL* (5), and in 77 patients the classical genetic fusions were not present and could not be identified by Multiplex PCR or through molecular sequencing (Table 1).

Our analysis showed significant differences between the age group and the genetic fusion presented (*p* < 0.0001), where it was observed that 26.8% and 9.3% of patients aged between 1 and 9 years were positive for *E2A-PBX1* and *TEL-AML1*, respectively. Between patients aged 10 years or more, 21.3% showed the *BCR-ABL* fusion and 6.4% the *SIL-TAL* translocation, and in patients younger than 1 year *MLL-AF4* were visualized in 33.3% of diagnoses. The majority of undiagnosed genetic fusions were found in patients aged 10 years or more (61.9%).

A significant difference was also found when Immunophenotype and white blood cells count were compared (*p* = 0.0002), showing that patients with biphenotypic and T-ALL cell immunophenotype presented white blood cells count (WBC) greater than 100 × 10^3^/mm^3^, while the B-ALL cell immunophenotype patients mostly had the count between 50–100 × 10^3^/mm^3^ (94.7%). When genetic fusions were compared to the WBC, we also found a significant statistics (*p* = 0.0002), additionaly the results showed that patients with *E2A-PBX1* and *TEL-AML1* had WBC between 50 × 10^3^/mm^3^ and 100 × 10^3^/mm^3^, as well as in patients with *BCR-ABL*, *MLL-AF4*, and *SIL-TAL* fusions, WBC were greater than 100 × 10^3^/mm^3^. Moreover, 57% of patients with no classical genetic fusion, presented WBC lower than 50 × 10^3^/mm^3^.

### 3.2. hTERT Shows Enhanced Gene Expression in ALL Pediatric Patients

**Figure 1:**
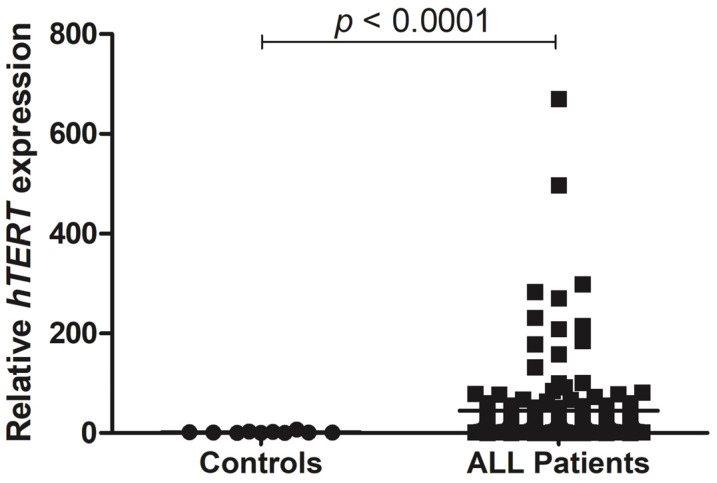
The ALL patients demonstrate enhanced *hTERT* expression. Gene expression analysis of *hTERT* in ALL blood samples from pediatric patients (*n* = 244). Relative *hTERT* expression was calculated using *ABL* and *GAPDH* as endogenous normalizers. Data are presented as the median and each dot plot represents the *hTERT* expression in a single patient. For statistical analysis, the normal distribution was confirmed by the Shapiro–Wilk normality test followed by the Mann–Whitney test. Comparison with control samples: *p* < 0.0001. ALL: acute lymphoblastic leukemia.

We performed an analysis of gene expression of telomerase (*hTERT*) in 244 blood samples from pediatric patients with ALL and compared them to blood samples of 10 healthy patients (control group). *hTERT* was approximately 26.7-fold change overexpressed in patients with ALL when compared to the control group (*p* < 0.0001) (Figure 1).

Next, we investigated if *hTERT* expression differed among the clinical features of pediatric ALL patients. For this purpose, data were normalized and categorized assuming Log2 fold change expression levels, and patients were divided in two main groups: with “low *hTERT* expression” represented by patients with fold change ≤ 5 and “high *hTERT* expression” comprise those patients with a fold change > 5.

**Figure 2:**
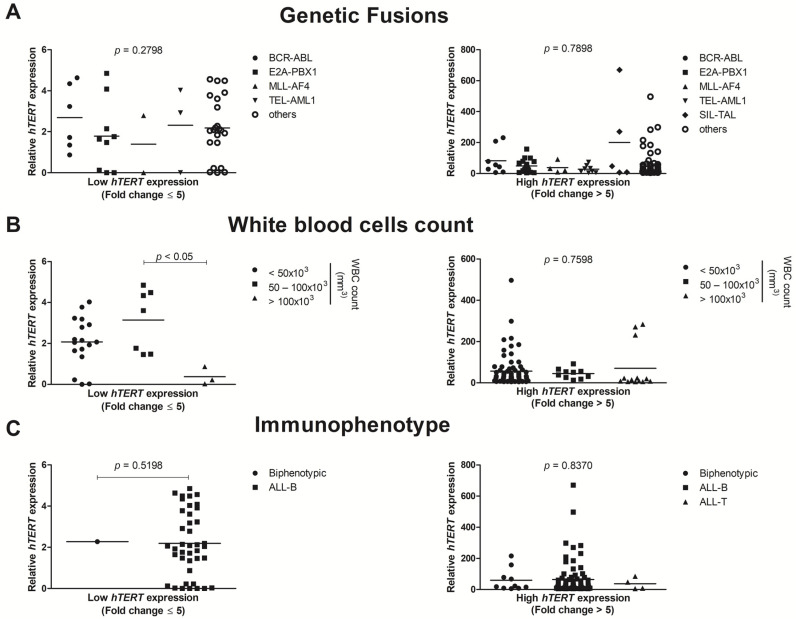
The ALL pediatric cohort (*n* = 148) was separated into two groups, with low (fold change ≤ 5) and high (fold change > 5) *hTERT* expression. (**A**) Those patients did not statisticaly differ despite all genetic fusions. (**B**) For WBC, only in the group with low *hTERT* expression, a significant difference was observed between 50–100 × 10^3^ mm^3^ and >100 × 10^3^ mm^3^ samples (*p* < 0.05) (**C**) Immunophenotype features did also not present significant results in low or high *hTERT* expression groups. Relative *hTERT* expression was calculated using *ABL* and *GAPDH* as endogenous normalizers. Data are presented as the median and each dot plot represents the *hTERT* expression in a single patient. For statistical analysis, the Shapiro–Wilk normality test was performed, followed by the Kruskal–Wallis test and Dunn’s Multiple Comparison post-test. WBC: white blood cells count, ALL: acute lymphoblastic leukemia.

As shown in Figure 2A, when we compared the *hTERT* gene expression between each genetic fusions: *E2A-PBX1*, *BCR-ABL*, *TEL-AML1*, *MLL-AF4*, *SIL-TAL*, and others (patients that did not presented any classical fusions), no statistically significant differences were found in low (*p* = 0.2798) or high (*p* = 0.7898) *hTERT* expression groups. When the patients were categorized by the white blood cells count (WBC) into <50 × 10^3^/mm^3^, 50–100 × 10^3^/mm^3^ and >100 × 10^3^/mm^3^, no statistical difference was seen in the group of high *hTERT* expression (*p* = 0.7598). However, in the low *hTERT* expression group, there was a significant statistical result between the groups of patients that presented a WBC range 50–100 × 10^3^/mm^3^ and >100 × 10^3^/mm^3^ (*p* < 0.05).

Moreover, Figure 2C shows that *hTERT* expression was not statistically different in ALL pediatric patients when sorted by immunophenotype (biphenotypic, ALL-B cell, and ALL-T cell) at low (*p* = 0.5198) or high (*p* = 0.8370) *hTERT* gene expression.

**Figure 3:**
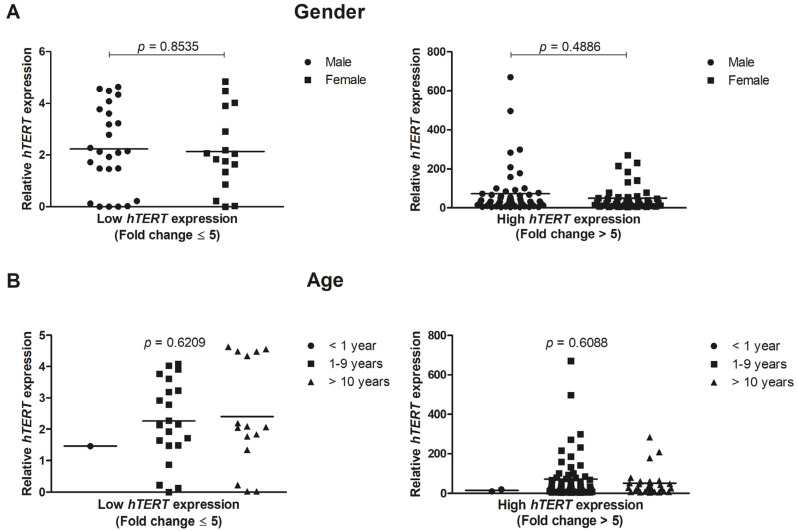
The ALL patients (*n* = 148) were separated into two groups, with low (fold change ≤ 5) and high (fold change > 5) *hTERT* expression. (**A**) Gender and (**B**) Age did not imply significant alterations between groups with low or high *hTERT* gene expression. Relative *hTERT* expression was calculated using *ABL* and *GAPDH* as endogenous normalizers. Data are presented as the median and each dot plot represents the hTERT expression in a single patient. For statistical analysis, the Shapiro–Wilk normality test was performed, followed by the Kruskal–Wallis test and Dunn’s Multiple Comparison post-test for age analysis or t-test for gender.

Figure 3A,B shows the *hTERT* expression in pediatric ALL patients sorted by gender and age (<1 year, 1–9 years and >10 years). In regards to the gender parameter, in low or high expression, no statistically significant differences were seen, between the analyzed groups (*p* = 0.8535, and *p* = 0.4886, respectively). Furthermore, for age analysis, no significant results were also found between groups of low (*p* = 0.6209) or high (*p* = 0.6088) *hTERT* expression.

### 3.3. Patient’s Overall Survival

**Figure 4:**
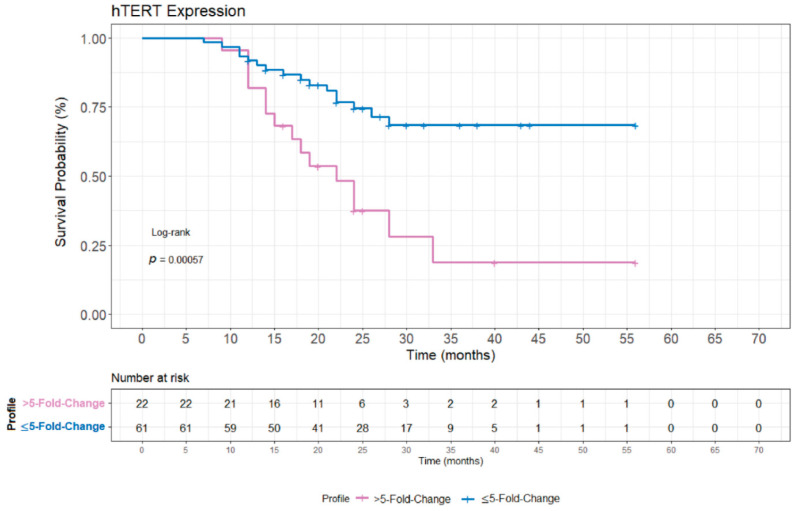
Comparison of survival time for patients with different levels of *hTERT* expression. Survival time was statistically different between patients. Patients expressing *hTERT* showed a significantly lower survival rate in patients (*p* < 0.0001) who had higher expression levels (>5 fold-change).

Patient’s overall survival rate (OS) was also performed to evaluate the role of *hTERT* gene expression levels and its potential association with prognosis. For this analysis, patients who presented all the clinical risk parameters used for the previous statistical analysis, were included to perform the overall survival rate. A total of 83 patients were included, and the median time of follow-up was 23.9 months (range 7–56 months). We performed the OS analysis using the same normalized expression data divided in two groups: patients who presented >5-fold change and ≤5-fold change gene expression levels. The ALL patients who presented *hTERT* expression levels higher than 5-fold change, presented a poorer prognosis with significantly lower survival rates (*p* < 0.0001), as shown in Figure 4.

### 3.4. PPI Network and Biological Functions of hTERT

**Figure 5:**
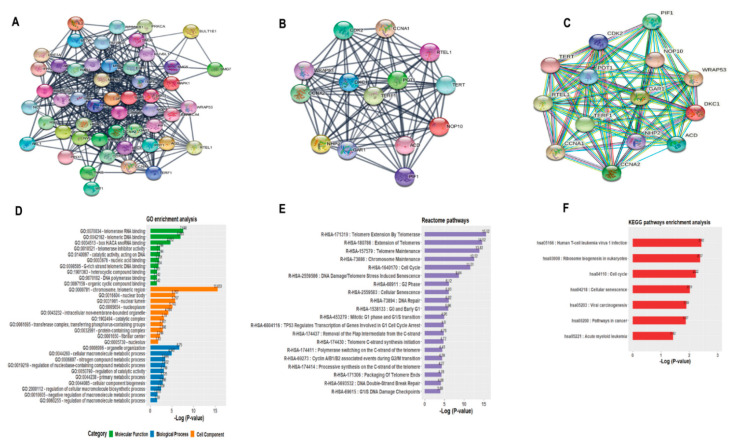
Protein–protein interaction network for *hTERT*. (**A**) Prototype of the PPI network built with Cytoscape containing 51 nodes and 377 edges. (**B**) PPI generated with MCODE presented a score of 13.53 with 14 nodes and 88 edges. (**C**) Validation done with String-db for the PPI topology containing 14 gene hubs that was predicted by the MCODE algorithm. (**D**) GO functional annotation for PPI genes. (**E**) Identification of enzymatic reaction pathways of REACTOME and (**F**) Pathways of KEGG. All annotation terms were normalized with Log10 (*p*-value). PPI: Protein–Protein Interaction.

To identify and highlight which cellular and molecular pathways the participation of *hTERT* would be most prominent, we proposed a computational assembly of a Protein-Protein Interaction (PPI) network. The PPI generated with MCODE presented a score of 13.53 with 14 nodes and 88 edges (Figure 5A–C). We validated this topology and achieved a PPI enrichment *p*-value < 1 × 10^−16^. We identified a total of 14 gene hubs: *ACD*, *CCNA1*, *CCNA2*, *CDK2*, *DKC1*, *GAR1*, *NHP2*, *NOP10*, *PIF1*, *PPOT1*, *PRTEL1*, *PTERF1*, *PTERT*, *PWRAP53*, which showed the highest number of significant interactions. Furthermore, the enrichment analysis of GO for the group with stonger *hTERT* expression, showed these genes strongly associated in the biological process, cellular component, and molecular function of important telomerase events, which were observed with strong enrichment for GO:0000781-chromosome, telomeric region, GO:0070034-telomerase RNA binding, GO:0042162-telomeric DNA binding (Figure 5D).

To provide a broader interpretation of the *hTERT* biological functions and interaction with these genes, we have identified 20 REACTOME pathways, highlighting telomere extension by telomerase, telomere extension, chromosome and telomerase maintenance, cell cycle (Figure 5E) that were highly correlated with broader KEGG pathways with emphasis on the cell cycle (hsa04110) (Figure 5F).

## 4. Discussion

Telomerase (*hTERT*) is expressed in stem cells that naturally undergo self-renewal processes to maintain their constant replication without losing portions of their telomeres. Such expression occurs in hematopoietic cells, proliferating lymphocytes, the degenerative basal layer of the epidermis of human skin and can also be detected in germ and embryonic cells due to their high replication rates. The altered expression of telomerase in humans leads to cell immortalization and it is a cancer hallmark [15,16,19,28,29,30].

In the present study, we demonstrate that *hTERT* is significant overexpressed in all the 244 ALL pediatric patients cohort analyzed in this study, in comparison to healthy controls (*p* < 0.0001), and, therefore, supporting more accurately the possibility of considering it as a molecular biomarker in this cancer model. Studies have shown that in ALL tumorigenesis model, the leukemic cells use the telomerase activity to first acquire an immortalized phenotype, preventing cellular apoptosis induced by the reduction of telomeres and then fomenting their ability of self-renewal [31,32,33,34].

We also performed a correlation analysis between *hTERT* expression, despite low or high expressions, and clinical risk assessment variables: gene fusions (*BCR–ABL*; *E2A–PBX1*; *MLL-AF4*; *TEL–AML1*; *SIL–TAL1* or other fusions), age and immunophenotyping, however, none of them alone was significantly associated with telomerase overexpression (*p* > 0.05). Similar findings were described in the literature regarding clinical data and telomeres/telomerase activity, showing that *hTERT* overexpression seems to have a prominent importance in this disease model, independently of clinical findings and variables of risk management [32,35,36,37].

In regards to leucometric parameters, patients with high *hTERT* expression did not present significant statistical results (*p* > 0.05) in any of the groups, however, the groups of patients with low *hTERT* expression and with a WBC ranges of 50–100 × 10^3^/mm^3^ and >100 × 10^3^/mm^3^, when compared, presented a significant statistical result (*p* < 0.05). This is a important finding, once high levels of WBC counts (>50 × 10^3^/mm^3^) are described as a desfavorable prognosis in clinical risk management guidelines [22,38], and this association with *hTERT* expression could provide an additional biomarker to clinical prognosis assessment.

This is the first study in Brazil and South America to correlate all clinical risk assessment variables with *hTERT* expression in ALL pediatric patients. Therefore, our data corroborate with a previous work by Ohyashiki et al. [39] who first demonstrate that an increased telomerase activity was associated with poorer survival in pediatric patients and these usually present higher telomerase activity than adults. Thus, the data presented here are a noteworthy finding that pointed a possible common molecular biomarker to manage ALL patients despite different clinical findings. That risk management of ALL pediatric patients take into consideration clinical variables is one of the most important information to prognosis assessment and to guide better therapeutical protocols in clinical trials and medical centers [1,40,41,42].

While literature demonstrate telomerase activity to be even more overexpressed in male ALL patients, when compared to female ones [43,44], the current study did not detect any statistically significant differences (*p* > 0.05) in *hTERT* expression levels among patients of different genders. In addition, the male: female ratio found in the present study was 1.3:1, which corroborates the results of another Brazilian study, in which the pediatric patients described presented a male: female ratio of 1.7:1 [40].

Chromosomal translocations are still the main marker of genetic instability in ALL leukemogenesis, the gene fusions t(1; 19)(q23; p13) that generates *E2A-PBX1* and t(9; 22)(q34; q11) that originates the chimeric gene *BCR-ABL*, were the two more frequent found in ALL cohort analyzed in this study, 20.27% and 12.83%, respectively. Several studies demonstrate that those two genetic fusions in particular, are the most therapeutic relevant in ALL clinical practice due to their poor prognosis and high-risk disease [1,45,46,47]. Nevertheless, most of ALL patients (52%) analyzed in this work did not present any classical gene fusions, described as main findings in this disease, which might compromise those patients to be treated with possible targeted therapies developed to specific genetical biomarkers, which have improved patient survival [48,49,50]. In this scenario, the presence of *hTERT* overexpression seen in those patients may arise as a potential therapeutical possibility [51,52].

In addition, the overall survival rate analysis demonstrated that patients who presented a higher *hTERT* expression (>5-fold change) had a poorer prognosis with significantly lower survival rates (*p* < 0.0001). Studies have pointed out that, telomeres and telomerase seem to have an important prognosis strength in different types of cancers and hematological malignancies [53,54,55,56,57]. Quantification of *hTERT* expression may assist when monitoring ALL minimal residual disease, serving as clinical follow-up for patients both in treatment and in complete remission after treatment [20,32,43].

Telomerase have been widely discussed as a potential antitumoral target in several haematological malignancies [37,58,59,60]. To identify *hTERT* biological interaction pathways in ALL pathogenesis, we constructed a computational PPI network, that demonstrate an important role for the *hTERT* involvement in pathways for chromosome maintenance, mitotic cell cycle transition, DNA double-strand break repair, cellular senescence, pathways in cancer and leukaemia. The generated PPI network also revealed a strong correlation between TERT and WRAP53 (WD40-enconding RNA antisense to p53), a protein that is widely involved in apoptosis, cell cycle regulation and the most important function is to act as an antisense regulator of p53 function and its dysfunction that could promote tumorigenesis [61,62,63]. Moreover, WRAP53 recently was found to be a telomerase holoenzyme subunit and present an essential role for telomere elongation, in addition, WRAP53 overexpression have been described in different human cancer cells, associated with a poorer prognosis suggesting that it might be an oncogene and as potential target for cancer therapy [64,65,66,67].

However, despite all scientific data reports, further studies needs to evaluate and explore, if both, the size of the telomeres and the telomerase gene expression, may assist in the investigation of hematological malignancies, other types of cancer and several diseases, as well as establishing telomerase as a potent molecular biomarker for diagnosis, prognosis, and targeted therapy [10,68,69].

## 5. Conclusions

The data presented in this study indicate that *hTERT* expression might seem to be linked to the pathogenesis of the different types of ALL found in the studied population, more precisely in the maintenance and disease persistence, a fact that may be understood in association to the multifactorial nature of leukemogenesis. In this context, *hTERT* expression emerges as an important molecular pathway in leukemogenesis regardless of the patient’s clinical variables; thus, the data here presented pointed it out as a valuable biomarker in pediatric acute lymphoblastic leukemia and a promising target for new therapeutic and prognostic measures.

## Figures and Tables

**Table 1:** Clinical features analysis of acute lymphoblastic leukemia patients.

N (%)											
	Age				WBC (×10^3^)/mm^3^				Gender		
	>1	1–9	≥10	*p*-Value	>50	50–100	>100	*p*-Value	M	F	*p*-Value
**Immunophenotype**				0.3853				0.0002 *			0.5586
**Biphenotypic**	0 (0)	11 (11.3)	2 (4.3)		10 (10)	1 (5.3)	2 (6.9)		7 (8.3)	6 (9.4)	
**T-ALL cell**	0 (0)	2 (2.1)	3 (6.4)		0 (0)	0 (0)	5 (17.2)		4 (4.8)	1 (1.6)	
**B-ALL cell**	4 (100)	84 (86.6)	42 (89.4)		90 (90)	18 (94.7)	22 (75.9)		73 (86.9)	57 (89.1)	
**Genetic Fusion**				<0.0001 **				0.0002 *			0.9998
***E2A-PBX1***	0 (0)	26 (26.8)	4 (8.5)		19 (19)	8 (42.1)	3 (10.3)		17 (20.2)	13 (20.3)	
***BCR-ABL***	1 (16.7)	8 (8.2)	10 (21.3)		12 (12)	1 (5.3)	6 (20.7)		11 (13.1)	8 (12.5)	
***TEL-AML1***	0 (0)	9 (9.3)	1 (2.1)		8 (8)	2 (10.5)	0 (0)		6 (7.1)	4 (6.3)	
***MLL-AF4***	2 (33.3)	5 (5.2)	0 (0)		4 (4)	1 (5.3)	2 (6.9)		4 (4.8)	3 (4.7)	
***SIL-TAL***	0 (0)	2 (2.1)	3 (6.4)		0 (0)	0 (0)	5 (17.2)		3 (3.6)	2 (3.1)	
**Others**	1 (16.7)	47 (48.5)	29 (61.7)		57 (57)	7 (36.8)	13 (44.8)		43 (51.2)	34 (53.1)	

Legend: *p*-value by Chi-square test; α = 0.05. F: female; M: male; WBC: White blood cell count. Statistically significant values: * *p* = 0.0002; ** *p* < 0.0001.

## Supplementary Materials

The following are available online at https://www.mdpi.com/article/10.3390/genes12101632/s1, Supplementary file 1: Table S1—Primers applied for gene fusion identification.
