# Supplementary material for: Telomerase (hTERT) Overexpression Reveals a Promising Prognostic Biomarker and Therapeutical Target in Different Clinical Subtypes of Pediatric Acute Lymphoblastic Leukaemia
Source: Genes (Basel). 2021 Oct 17;12(10):1632. doi: 10.3390/genes12101632 (PMC8535500; doi:10.3390/genes12101632)
Supplement: Supplementary file 1 [file genes-12-01632-s001.zip › genes-1403362-supplementary.pdf]

TABLE S1 – PRIMERS APPLIED FOR GENE FUSION IDENTIFICATION

Genes	Primers (5´-3´)	Size (pb)	Position	Exons
E2A	CTACTCCCCGGATCACTCAA	20	1086 a 1105	13
PBX1	AGGCTTCATTCTGTGGCAGT	20	3893 a 3912	2
MLL	CGCCCAAGTATCCCTGTAAA	20	4071 a 4090	8
AF4	GAGCATGGATGACGTTCCTT	20	1546 a 1565	8
BCR	TCGCAGAACTCGCAACAGT	19	1707 a 1725	1
ABL	ACACCATTCCCCATTGTGAT	20	284 a 303	3
TEL	TCTCTCATCGGGAAGACCTG	20	1191 a 1210	5
AML1	TGCGGTAGCATTTCTCAGC	19	619 a 637	5
SIL	TCCTACCCTGCAAACAGACC	20	73 a 92	1
TAL	AGGCGGAGGATCTCATTCTT	20	1250 a 1269	4
